# Supplementary material for: Bafilomycin A1 Inhibits HIV-1 Infection by Disrupting Lysosomal Cholesterol Transport
Source: Viruses. 2024 Aug 29;16(9):1374. doi: 10.3390/v16091374 (PMC11435809; doi:10.3390/v16091374)

Figure S1: Effects of bafilomycin A1 on HIV-1 replication (time course). Jurkat cells were infected with HIV-1_{NL4-3} (4 ng of p24/2x10^5 cells) in the absence or presence of bafilomycin A1 (5 nM). Virus replication (A) and cell viability (B) were measured at 4, 6, and 8 days post-infection using ELISA (p24) and MTS assay, respectively. Values for cell viability are relative to the DMSO treatment control (100%). Data are mean ± SD (n=3). *** $p<0.001$. ns, non-significant.

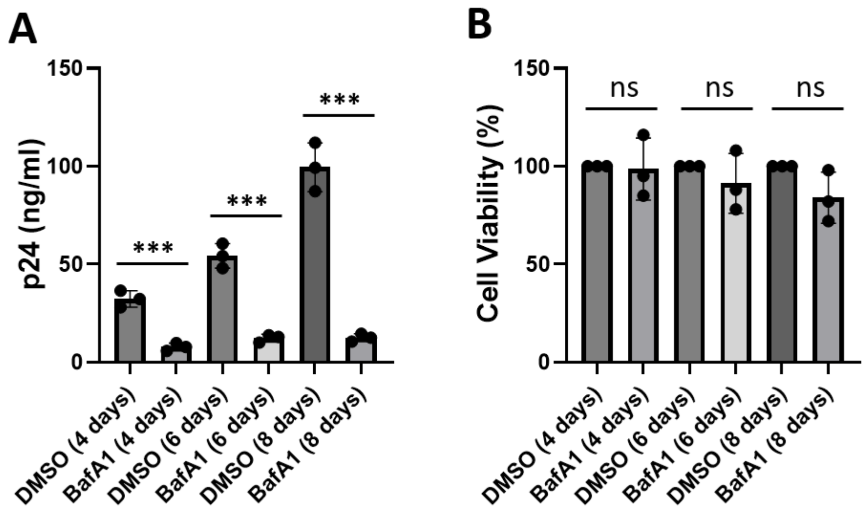

Figure S2: Bafilomycin A1 causes an accumulation of cholesterol in lysosomes in HIV-1-infected cells. TZB-bl cells were infected with HIV-1 in the absence or presence of bafilomycin A1 (5 nM) for 48 hours. Unesterified cholesterol was detected using filipin. Lysosomes were detected using anti-LAMP1 antibody with CF488-conjugated secondary antibody. Images (A) (60x) were obtained using the Nikon A1R confocal microscope. Scale bar = 20 µm. Images are representative of three independent experiments. Pearson's correlation coefficient (B) of filipin and LAMP1 were measured from 60 cells in 4 different fields. ** *p*<0.01.

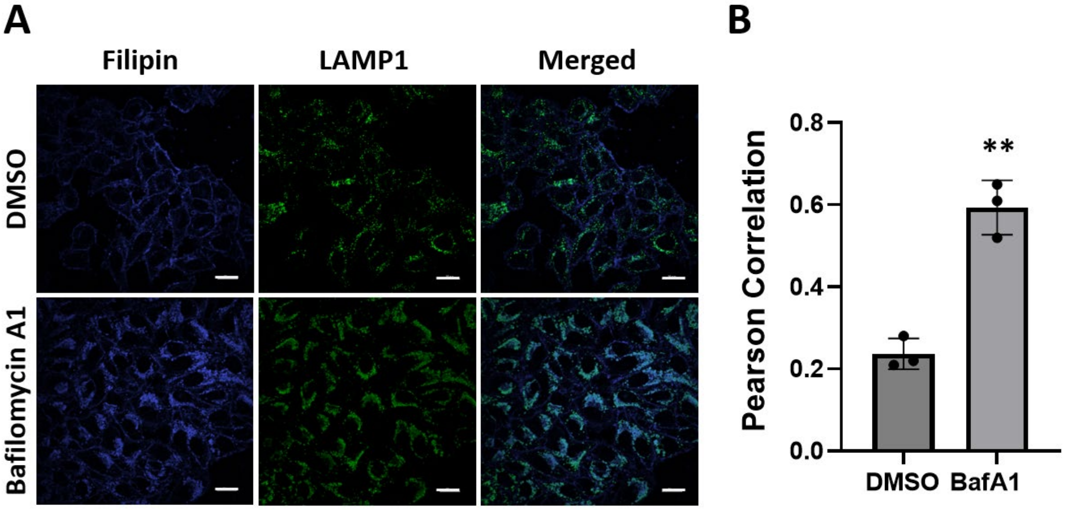

Figure S3: Bafilomycin A1 disrupts the autophagic pathway. TZM-bl cells were treated with DMSO or bafilomycin A1 (1 or 5 nM) for 48 h. Western blot was conducted to detect LC3-I, LC3-II, and GAPDH. Images (**A**) are a representative of three independent experiments. The band intensities were quantified using the Image J software. The ratio of LC3-I/GAPDH (**B**), LC3-II/GAPDH (**C**), and LC3-II/LC3-I (**D**) is shown. Data are mean ± SD (n=3). *** p<0.001.

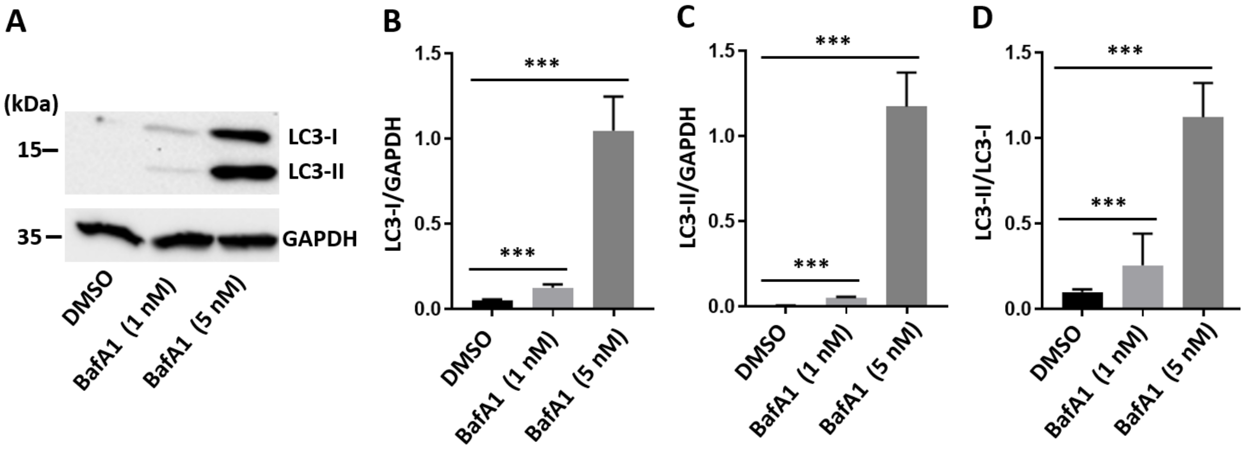

Figure S4: Uncropped Western blot images

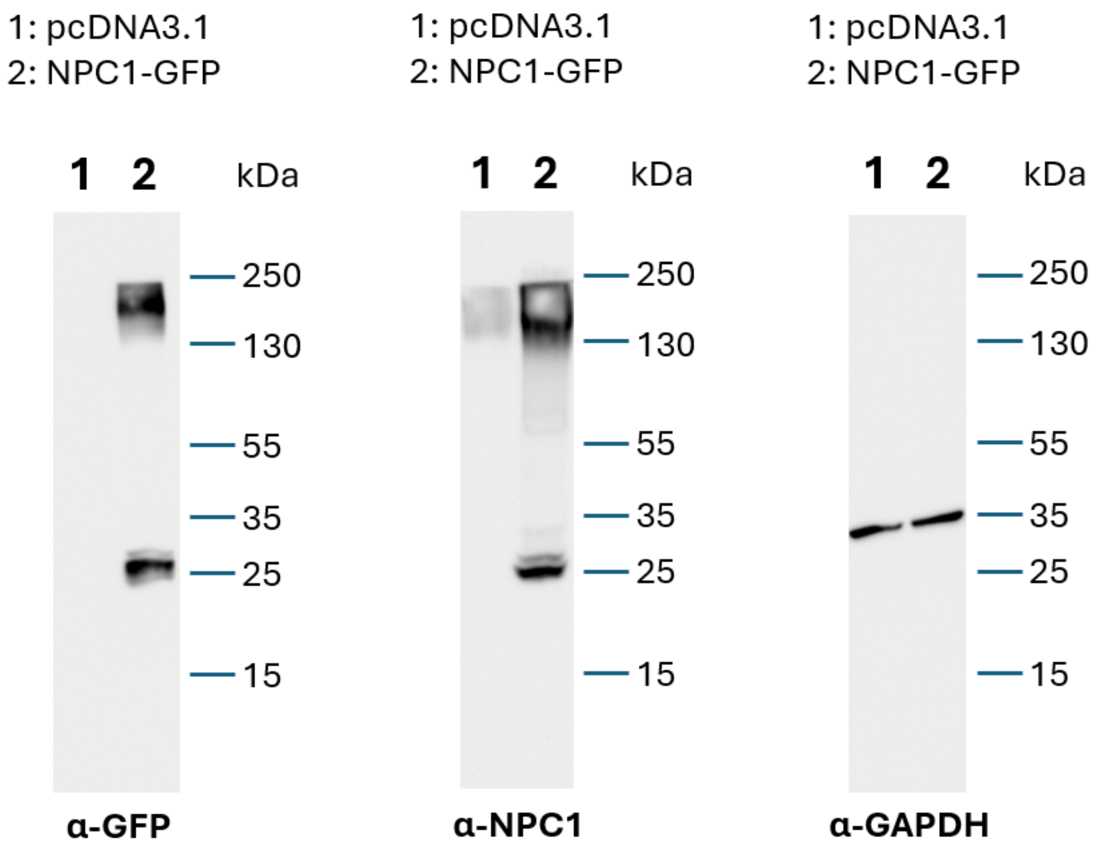

Supplement: Supplementary file 1 [file viruses-16-01374-s001.zip › viruses-3100038-supplementary.pdf]
